# Using Matrix-Assisted Laser Desorption Ionization-Time of Flight (MALDI-TOF) Complemented with Selected 16S rRNA and *gyrB* Genes Sequencing to Practically Identify Clinical Important Viridans Group Streptococci (VGS)

**DOI:** 10.3389/fmicb.2016.01328

**Published:** 2016-08-26

**Authors:** Menglan Zhou, Qiwen Yang, Timothy Kudinha, Li Zhang, Meng Xiao, Fanrong Kong, Yupei Zhao, Ying-Chun Xu

**Affiliations:** ^1^Department of Clinical Laboratory, Peking Union Medical College Hospital, Chinese Academy of Medical SciencesBeijing, China; ^2^Graduate School, Peking Union Medical College, Chinese Academy of Medical SciencesBeijing, China; ^3^School of Biomedical Sciences, Charles Sturt UniversityOrange, NSW, Australia; ^4^Centre for Infectious Diseases and Microbiology Laboratory Services, Westmead HospitalWestmead, NSW, Australia; ^5^Department of General Surgery, Peking Union Medical College Hospital, Chinese Academy of Medical SciencesBeijing, China

**Keywords:** *Streptococcus*, viridans group streptococci (VGS), matrix-assisted laser desorption ionization-time of flight (MALDI-TOF), 16S rRNA gene, *gyrB* gene

## Abstract

There are challenges in viridans group streptococci (VGS) identification especially for the mitis group. Few studies have investigated the performance of MALDI-TOF MS system in VGS identification. Using 16S rRNA gene and *gyrB* gene sequencing as a gold standard, the performance of two MALDI-TOF MS instruments in the identification of 181 VGS clinical isolates was studied. The Bruker Biotyper and Vitek MS IVD systems correctly identified 88.4% and 98.9% of the 181 isolates, respectively. The Vitek MS RUO system was the least reliable, only correctly identifying 38.7% of the isolates to species level with several misidentifications and invalid results. The Bruker Biotyper system was very unreliable in the identification of species within the mitis group. Among 22 non-pneumococci isolates (*S. mitis*/*S. oralis*/*S. pseudopneumoniae*), Biotyper misidentified 21 of them as *S. pneumoniae* leading to a low sensitivity and low positive predictive value in these species. In contrast, the Vitek MS IVD demonstrated a better resolution for pneumococci and non-pneumococci despite the inability to distinguish between *S. mitis*/*S. oralis*. For more accurate species-level identification, further improvements in the VGS spectra databases are needed. Based on MALDI-TOF analysis and selected 16S rRNA gene plus *gyrB* genes sequencing, we designed a practical VGS identification algorithm.

## Introduction

The viridans group streptococci (VGS) are a heterogeneous group of gram positive cocci, which form part of the normal human flora of the oral cavity, respiratory, urogenital, and gastrointestinal tracts (Spellberg and Brandt, 2011). Currently, VGS is subdivided into six major groups: *S. anginosus, S. bovis, S. mitis, S. mutans, S. salivarius*, and *S. sanguinis* (Facklam, 2002; Doern and Burnham, 2010). Accurate identification of species within the VGS group is important for assessing the clinical significance of the organism and to facilitate appropriate antimicrobial therapy (Sinner and Tunkel, 2009; Doern and Burnham, 2010). However, due to constant taxonomic changes in the VGS group, identification of species is challenging. No phenotypic identification method can be used as a gold standard for VGS as most methods, including API Strep, and Vitek 2, have only 30–80% identification accuracy, depending on the species (Ikryannikova et al., 2011; Teles et al., 2011).

Sequence analysis targeting different single genes such as 16S rRNA gene, *rpoA, rpoB, rnpB, rodA, soda*, and *gdh*, have been used in the identification of VGS species with various degrees of success (Poyart et al., 1998; Ip et al., 2006; Westling et al., 2008; Konishi et al., 2009; Nielsen et al., 2009; Park et al., 2010). Currently, only multilocus sequence analysis (MLSA) can accurately and reliably identify species within the VGS group. But MLSA is too expensive and laborious for routine laboratory diagnostic use (Bishop et al., 2009). Recently, Galloway-Peña et al. reported that the *gyrB* amino acid sequence may offer a more practical and accurate method for speciating invasive VGS strains than MLSA (Galloway-Pena et al., 2014).

In recent years, matrix-assisted laser desorption ionization-time of flight (MALDI-TOF) has emerged as a rapid and cost-effective alternative assay for bacterial identification (Seng et al., 2009; Bizzini et al., 2010; Neville et al., 2011). Nevertheless, some studies indicate that this assay has problems in distinguishing species within the *S. mitis* group (Ikryannikova et al., 2011; Davies et al., 2012; Wessels et al., 2012).

We investigated the performance of two MALDI-TOF MS systems, namely Bruker Biotyper (Daltonics, German) and Vitek MS (bioMérieux, France) in the identification of species within the VGS group, using sequencing of the 16S rRNA, and *gyrB* genes as a gold standard.

## Materials and methods

### Bacterial strains and cultures

Clinically significant VGS isolates (*n* = 181) from sputum (*n* = 81), blood cultures (*n* = 29), tracheal aspirates (*n* = 11), midstream urine (*n* = 9), and other various sterile and non-sterile sites (*n* = 51) from Peking Union Medical College Hospital (PUMCH; 2013–2014), were studied (Supplementary Table S1). Initial identification of these isolates was done by conventional methods (positive Gram stain, coccus morphology in chains, alpha-hemolysis, and a negative catalase test) and Vitek 2 compact system. Optochin and bile solubility tests were performed to differentiate pneumococci from non-pneumococci. The isolates were stored at −70°C in skim milk until further testing.

### Gene sequencing-based identification

Template DNA was prepared as described by. Dubois et al. (2013). The16S rRNA gene was amplified for all the isolates using the universal primers 27F 5′-AGAGTTTGATCMTGGCTCAG-3′ and 1492R 5′-TACGGYTACCTTGTT ACGACTT-3′ (Seng et al., 2009). Purified PCR products and sequencing primers (the same as for amplification) were mixed and sent to Ruibiotech (Beijing, China) for sequencing. Species identification was performed by comparing the obtained sequences against those in the GenBank database using BLASTn (www.ncbi.nlm.nih.gov/blast). A sequence similarity of 99% was applied as species identification “cut-off” value for the 16S rRNA gene region. Amplification and sequencing of the *gyrB* gene encoding DNA Gyrase subunit B was performed for all the non-pneumococci using primers gyrB7F 5′-GAAGTDGTIAARATYACBAAY CG-3′ and gyrB5R 5′-ACATCDGCATCRGTCAT-3′ (Maeda et al., 2011). Both the *gyrB* nucleotide and amino acid sequences were analyzed through BLASTn and BLASTp and a nucleotide sequence similarity of 96% or a signature amino acid at a certain position was applied as species identification standard according to Galloway-Pena et al. (2014). Besides, the phylogenetic trees were generated based on 16S rRNA gene and *gyrB* gene. The region used for phylogenetic analyses and species identification was nucleotides 86–1336 for 16S rRNA gene and nucleotides 1113–1512, corresponding to amino acids 371–503 for *gyrB* gene respectively. Following alignment with Clustal W, the sequences were analyzed in MEGA version 5.2 to create radial trees using the neighbor-joining statistical method and the maximum likelihood composite model.

### MALDI-TOF analysis

Two MALDI-TOF MS systems, Bruker Biotyper and Vitek MS, including both the *In Vitro* Diagnosis (IVD) and Research Use Only (RUO) modes were used to identify the 181 VGS isolates, according to the manufacturer's instructions. For both MALDI-TOF MS systems, direct transfer method were used for sample preparation. A small portion of a single colony after 24 or 48 h of incubation was smeared onto a target plate using a wooden cocktail stick, and covered with 1 μl matrix solution (α- cyano-4-hydroxycinnamic acid in 50 acetonitrile/2.5% trifluoroacetic acid, Bruker Daltonics, Bremen, Germany; α-cyano-4-hydroxycinnamic acid, VITEK® MS CHCA) immediately. For Bruker Biotyper, measurements were performed with the Bruker Biotyper MALDI-TOF MS system using FlexControl software with Compass Flex Series version 1.3 software and a 60-Hz nitrogen laser (337 nm wavelength). Spectra ranging from 2000 to 20,000 m/z were analyzed using the MALDI Biotyper system's automation control and the current Bruker Biotyper V.3.3.1.2 software and library [database (DB) 5989 with 5989 entries]. For Vitek MS, measurement was performed using the manufacturer's suggested settings using automated collecting spectra. Captured spectra (mass range of 2–20 kDa in the linear mode) were analyzed using the MALDI-TOF MS IVD MYLA database v2.0 and also the MALDI-TOF MS RUOsystem with the SARAMIS™ database v4.10 of bioMérieux. All identifications displaying a single result with a confidence score ≥1.7 or a confidence value of 99.9% were considered acceptable for Bruker Biotyper MS and Vitek MS, respectively (Karpanoja et al., 2014). For both MS systems, all isolates yielding a single result without acceptable confidence level or multiple results or “no identification” results were re-tested. If a single, species-level identification was obtained upon repeat analysis, this identification was considered to be the final MS result; otherwise no further analysis was performed.

### Statistical analysis

Agreement and validity values were calculated with a 95% confidence interval (CI) based on an exact binomial distribution. Data were analyzed using SPSS, version 15.0 (SPSS Inc, Chicago, IL, USA).

## Results

### The 16S rRNA gene and *GyrB* gene sequencing-based identification

**Figure 1:**
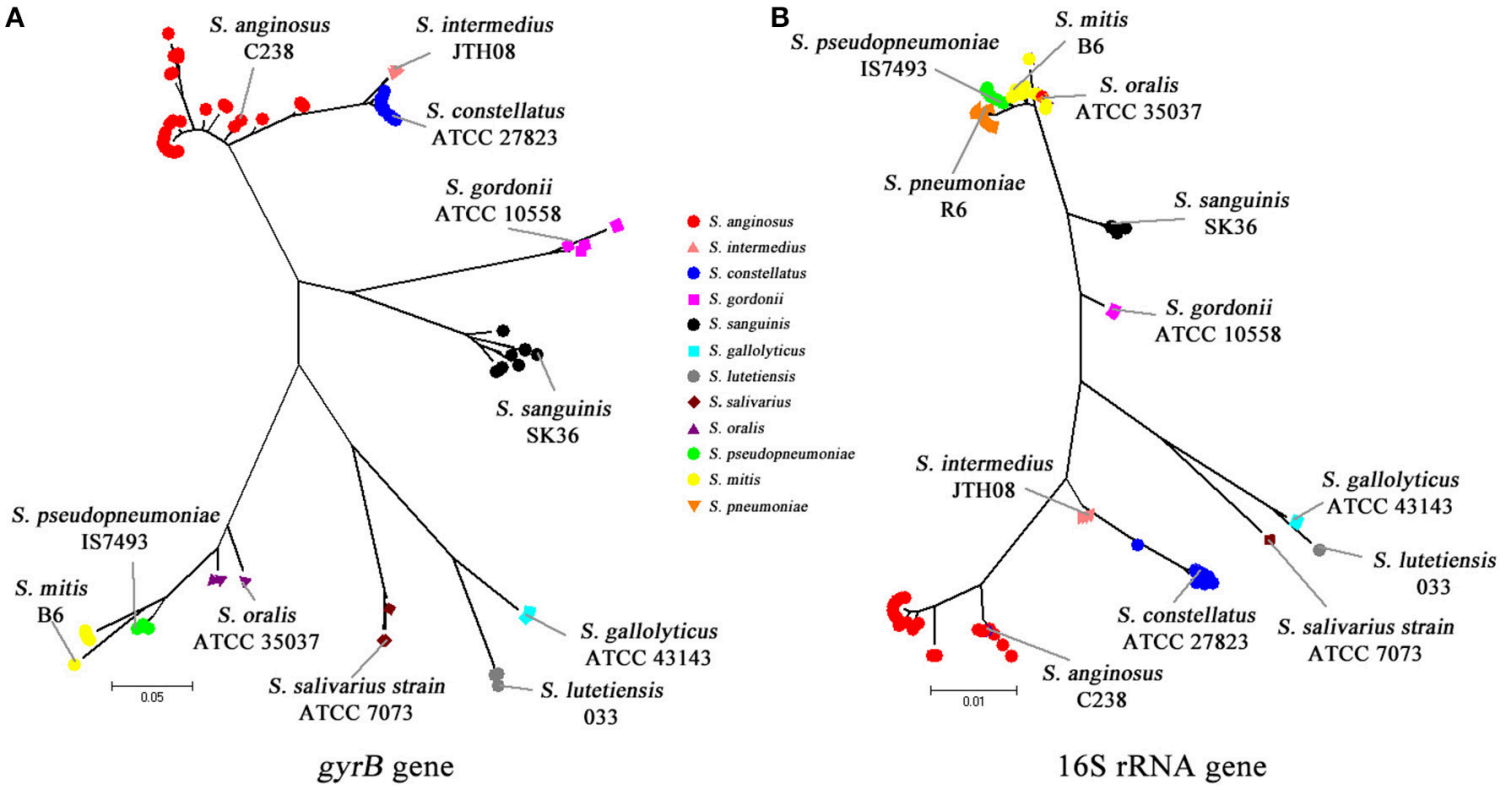
**Phylogenetic analyses using 16S rRNA and gyrB fragment sequences. (A)** Phylogenetic analysis using the gyrB sequence from nucleotides 1113–1512. **(B)** Phylogenetic analysis using the 16S rRNA sequence from nucleotides 86 to 1336.

Using a combination of 16S rRNA gene and *gyrB* gene sequencing, all the 181 VGS isolates were assigned to species level on the basis of a ≥99 and ≥96% gene sequence similarity with published sequences in the GenBank. The DNA sequences of the16S rRNA gene and *gyrB* gene of the 181 VGS isolates studied have been submitted to GenBank database (accession numbers of KX661043–KX661223 for 16S rRNA gene and KX661224–KX661319 for *gyrB* gene respectively (Supplementary Table S2). Figure 1 shows the phylogenetic trees generated by *gyrB* gene and 16S rRNA gene. As is shown in Figure 1A, *gyrB* nucleotide sequence successfully delineated all of the 96 non-pneumococci strains into individual species branches while in Figure 1B, all of the *S. oralis* isolates were branched with *S. mitis* isolates by 16S rRNA sequences. Besides, the bootstrap values between the mitis group were rather low compared to *gyrB* gene, which emphasizs the difficulties of using 16S gene sequences to correctly assign VGS strains to particular species. The identification results of the 181 VGS isolates are summarized in Supplementary Tables S3, S4, and S5, using gene sequencing as the gold standard.

A high level of similarity in the 16S rRNA gene sequences of *S. pneumoniae, S. pseudopneumoni*ae, *S. mitis*, and *S. oralis*, which could make it difficult in distinguishing these species, has been reported (Kawamura et al., 1995; Arbique et al., 2004; Suzuki et al., 2005; Haanpera et al., 2007). Nevertheless, a recent study by Scholz et al. demonstrated that *S. pneumoniae* has a cytosine at position 203 of the 16S rRNA gene, while all other mitis group streptococci have adenine in that position (Scholz et al., 2012). In this study, we also checked position 203 of the 16S rRNA gene sequences, and confirmed that 85 isolates were pneumococci and the other 96 isolates non-pneumococci.

### MALDI-TOF analysis

Compared to the gold standard, the Bruker Biotyper system correctly identified 88.4% (160/181) of the isolates to species level and misidentified 11.6% (21/181) of the isolates. This system performed poorly in the identification of species in the mitis group, correctly identifying 80.4% (86/107) of the isolates to species level and misidentifying 19.6% (21/107) of the isolates. Optochin tests were performed on all isolates with an identification result of *S. pneumoniae* in an atmosphere of 5% CO_2_, among which 85 isolates were sensitive and the other 21 were resistant. Specifically, 100 (11/11), 100 (2/2), and 88.9% (8/9) of *S. mitis, S. oralis*, and *S. pseudopneumoniae* isolates were misidentified as *S. pneumoniae*, respectively. For all other groups, the Bruker Biotyper system performed excellently, accurately identifying all the species within each of the respective groups. Furthermore, the system did not yield an invalid or “no identification result” on any isolates (Supplementary Table S3).

Vitek MS IVD correctly identified 98.9 (179/181) of the VGS isolates to species level, 0.55 (1/181) to group level, and 0.55% (1/181) with no identification. In contrast to the Bruker MS system which performed dismally in the identification of *S. pseudopneumoniae* isolates, the Vitek MS IVD correctly identified the majority (8 of 9, 88.9%) of these isolates to species level and the remaining one isolate to group level (Supplementary Table S4). Furthermore, the Vitek MS IVD gave a “no identification” result on one isolate within the bovis group (*S. gallolyticus*).

In comparison to Bruker Biotyper and Vitek MS IVD, the Vitek MS RUO system performed very poorly in VGS identification, with an overall correct species level, group level, and genus level identification rate of 38.7 (70/181), 42.0 (76/181), and 7.2% (13/181) respectively (Supplementary Table S5). The system especially performed poorly in identifying species within the mitis and bovis groups, with only 15.9 (17/107) and 25% (2/8) of the isolates correctly identified, albeit low number of isolates. In addition, 8.8 (16/181) and 3.3% (6/181) of the isolates studied were misidentified and yielded a “no identification” result, respectively.

### Three MALDI-TOF systems comparisons

Sensitivity, specificity, positive predictive value (PPV) and negative predictive value (NPV) of the three databases are shown in Table 1. Overall, Bruker Biotyper gave a 100% sensitivity for the majority of the species identified, except for the mitis group species, namely *S. mitis* (0%), *S. oralis* (0%), and *S. pseudopneumoniae* (11.1%), most of which were misidentified as *S. pneumoniae* (21 of 22, 95.6%). Furthermore, PPV was incalculable for *S. mitis* and *S. oralis* as none of these isolates were correctly identified by the Bruker Biotyper. In contrast to other species in the mitis group, *S. pneumoniae*, had a 100% (95 CI: 95.8–100.0%) sensitivity and a 100% (95 CI: 95.5–100.0%) NPV, while the specificity and PPV were relatively low, at 78.1% (95 CI:68.5–85.9%) and 80.2% (95 CI:71.3%–87.3%), respectively.

**Table 1:** **Identification performance comparison of three MALDI-TOF MS systems for each group of viridans group streptococci (VGS)**.

	**No. (%) of isolate**	**Bruker Biotyper MS system**				**Vitek MS IVD system**				**Vitek MS RUO system**			
		**Se (%)**	**Sp (%)**	**PPV (%)**	**NPV (%)**	**Se (%)**	**Sp (%)**	**PPV (%)**	**NPV (%)**	**Se (%)**	**Sp (%)**	**PPV (%)**	**NPV (%)**
**Mitis group**	**107**	**80.4** **(71.6–87.4)**	**71.6** **(60.0–81.5)**	**80.4** **(71.6–87.4)**	**71.6** **(60.0–81.5)**	**98.1** **(93.4–99.8)**	**98.7** **(92.7–100)**	**99.1** **(94.9–100)**	**97.3** **(90.7–99.7)**	**15.0** **(8.8–23.1)**	**33.8** **(23.2–45.7)**	**24.6** **(14.8–36.9)**	**21.6** **(14.6–30.2)**
*S. mitis*	11	0.0 (0.0–28.5)	100 (97.9–100)	IC	93.9 (89.4–96.9)	100 (75.3–100)	99.4 (96.7–100)	92.9 (66.1–99.8)	100 (97.8–100)	0.0 (0.0–28.5)	95.3 (90.9–98.0)	0.0 (0.0–36.9)	93.6 (88.9–96.8)
*S. oralis*	2	0.0 (0.0–84.2)	100 (98.0–100)	IC	98.9 (96.1–99.9)	100 (75.3–100)	99.4 (96.7–100)	92.9 (66.1–99.8)	100 (97.8–100)	0.0 (0.0–84.2)	91.6 (86.6–95.2)	0.0 (0.0–21.8)	98.8 (95.7–99.9)
*S.pseudopneumoniae*	9	11.1 (0.3–48.3)	100 (97.9–100)	100 (2.5–100)	95.6 (92.6–98.6)	77.8 (40.0–97.2)	100 (97.9–100)	100 (59.0–100)	98.9 (95.9–99.9)	0.0 (0.0–33.6)	95.9 (91.8–98.4)	0.0 (0.0–41.0)	94.8 (90.4–97.6)
*S. pneumoniae*	85	100 (95.8–100)	78.1 (68.5–85.9)	80.2 (71.3–87.3)	100 (95.2–100)	100 (95.8–100)	100 (96.2–100)	100 (95.8–100)	100 (96.2–100)	18.8 (11.2–28.8)	80.2 (70.8–87.6)	45.7 (28.8–63.4)	52.7 (44.3–61.1)
**Anginosus group**	**52**	**100** **(93.2–100)**	**100** **(97.2–100)**	**100** **(93.2–100)**	**100** **(97.2–100)**	**100** **(93.2–100)**	**100** **(97.2–100)**	**100** **(93.2–100)**	**100** **(97.2–100)**	**93.1** **(59.0–84.4)**	**89.9** **(83.4–94.5)**	**74.5** **(60.4–85.7)**	**89.2** **(82.6–94.0)**
*S. anginosus*	29	100 (88.1–100)	100 (97.6–100)	100 (88.1–100)	100 (97.6–100)	100 (88.1–100)	100 (97.6–100)	100 (88.1–100)	100 (97.6–100)	100 (88.1–100)	91.5 (85.8–95.4)	69.1 (52.9–82.4)	100 (97.4–100)
*S. constellatus*	19	100 (82.4–100)	100 (97.8–100)	100 (82.4–100)	100 (97.8–100)	100 (82.4–100)	100 (97.8–100)	100 (82.4–100)	100 (97.8–100)	26.3 (9.2–51.2)	100 (97.8–100)	100 (47.8–100)	92.1 (87.0–95.6)
*S. intermedius*	4	100 (39.8–100)	100 (97.9–100)	100 (39.8–100)	100 (97.9–100)	100 (39.8–100)	100 (97.9–100)	100 (39.8–100)	100 (97.9–100)	100 (39.8–100)	100 (97.9–100)	100 (39.8–100)	100 (97.9–100)
**Sanguinis group**	**12**	**100** **(73.5–100)**	**100** **(97.8–100)**	**100** **(73.5–100)**	**100** **(97.8–100)**	**100** **(73.5–100)**	**100** **(97.8–100)**	**100** **(73.5–100)**	**100** **(97.8–100)**	**91.7** **(61.5–99.8)**	**100** **(97.8–100)**	**100** **(71.5–100)**	**99.4** **(96.8–100)**
*S. sanguinis*	8	100 (63.1–100)	100 (97.9–100)	100 (63.1–100)	100 (97.9–100)	100 (63.1–100)	100 (97.9–100)	100 (63.1–100)	100 (97.9–100)	87.5 (47.4–99.7)	100 (97.9–100)	100 (59.0–100)	99.4 (96.8–100)
*S. gordonii*	4	100 (39.8–100)	100 (97.9–100)	100 (39.8–100)	100 (97.9–100)	100 (39.8–100)	100 (97.9–100)	100 (39.8–100)	100 (97.9–100)	100 (39.8–100)	100 (97.9–100)	100 (39.8–100)	100 (97.9–100)
**Salivarius group**	**2**	**100** **(15.8–100)**	**100** **(98.0–100)**	**100** **(15.8–100)**	**100** **(98.0–100)**	**100** **(15.8–100)**	**100** **(98.0–100)**	**100** **(15.8–100)**	**100** **(98.0–100)**	**100** **(15.8–100)**	**100** **(98.0–100)**	**100** **(15.8–100)**	**100** **(98.0–100)**
*S. salivarius*	2	100 (15.8–100)	100 (98.0–100)	100 (15.8–100)	100 (98.0–100)	100 (15.8–100)	100 (98.0–100)	100 (15.8–100)	100 (98.0–100)	100 (15.8–100)	100 (98.0–100)	100 (15.8–100)	100 (98.0–100)
**Bovis group**	**8**	**100** **(63.1–100)**	**100** **(97.9–100)**	**100** **(63.1–100)**	**100** **(97.9–100)**	**87.5** **(47.4–99.7)**	**100** **(97.9–100)**	**100** **(59.0–100)**	**99.4** **(96.8–100)**	**25.0** **(3.2–65.1)**	**100** **(97.9–100)**	**100** **(15.8–100)**	**96.7** **(92.9–98.8)**
*S. lutetiensis*	2	100 (15.8–100)	100 (98.0–100)	100 (15.8–100)	100 (98.0–100)	100 (15.8–100)	100 (98.0–100)	100 (15.8–100)	100 (98.0–100)	0.0 (0.0–84.2)	100 (98.0–100)	IC	98.9 (96.1–99.9)
*S. gallolyticus*	6	100 (54.1–100)	100 (97.9–100)	100 (54.1–100)	100 (97.9–100)	83.3 (35.9–99.6)	100 (97.9–100)	100 (47.8–100)	99.4 (96.8–100)	33.3 (4.3–77.7)	100 (97.9–100)	100 (15.8–100)	97.8 (94.4–99.4)

*Se, sensitivity; Sp, specificity; PPV, positive predictive value; NPV, negative predictive value, IC, incalculable*.

*Group values of Se, Sp, PPV and NPV are in bold formats*.

Compared with Bruker Biotyper, Vitek MS IVD gave a better resolution for *S. mitis*/*S. oralis* identification with a sensitivity of 100% (95 CI: 75.3–100%) though the two species couldn't be distinguished from each other due to database limitation. Similarly, the overall sensitivity for *S. pseudopneumoniae* identification [77.8% (95 CI: 40.0–97.2%)] was higher than that of Bruker Biotyper [11.1% (95 CI: 0.3–48.3%)].

Expectedly, Vitek MS RUO showed low sensitivity for the identification of most species, except for *S. anginosus, S. intermedius, S. sanguinis, S. gordonii*, and *S. salivarius*.

## Discussion

Currently, only three studies have compared the performance of Bruker Biotyper vs. Vitek MS for VGS identification, with two of the studies analyzing a limited number of blood culture isolates (Karpanoja et al., 2014; Angeletti et al., 2015; Isaksson et al., 2015). In two of the studies, 16S rRNA gene and *rpoB* gene sequencing were used as a gold standard, while the third study didn't have a gold standard. Although the species/group distributions were different in each of these three studies, they all concluded that apart from the misidentification of other species as *S. pneumoniae* by Bruker Biotyper, the MALDI-TOF technique offers a reliable, rapid and cost saving method for VGS identification.

We evaluated the performance of two MALDI-TOF MS systems in VGS identification with a larger number of VGS species and a wider sample type base, using a combination of 16S rRNA gene and *gyrB* sequencing as a gold standard. Overall, the Vitek MS IVD system performed better than the Bruker Biotyper, accurately identifying 98.9% of the 181 VGS isolates, compared to 88.4% for Bruker Biotyper (*P* < 0.05). The lower overall performance of the Bruker Biotyper MS was due to misidentification of species within the mitis group, with 21 non-pneumococcal isolates misidentified as *S. pneumoniae*, which is in agreement with previous studies (Ikryannikova et al., 2011, 2013; Lopez Roa et al., 2013). Notably, we evaluated for the first time, the performance of Vitek MS RUO system though the results were not satisfying. However, this database covers rare VGS species not included in the Vitek MS IVD system (Karpanoja et al., 2014).

The superior performance of Vitek MS IVD in distinguishing *S. pneumoniae* from other mitis group species may be due to use of bin-weighting algorithms in species identification. The system identifies significant peaks of sample isolates and divides them into bins that are weighted according to frequency within a given species. Then the sums of bin weights are calculated using the advanced spectrum classifier algorithm to determine the best match, which may enhance sensitivity (Rychert et al., 2013). In contrast, Bruker Biotyper uses a single reference strain to generate multiple spectra and chooses a consensus spectra based on the reference spectra. After this, matching signals of the sample spectra are compared to the reference spectra and a score value is created (Welker, 2011).

**Figure 2:**
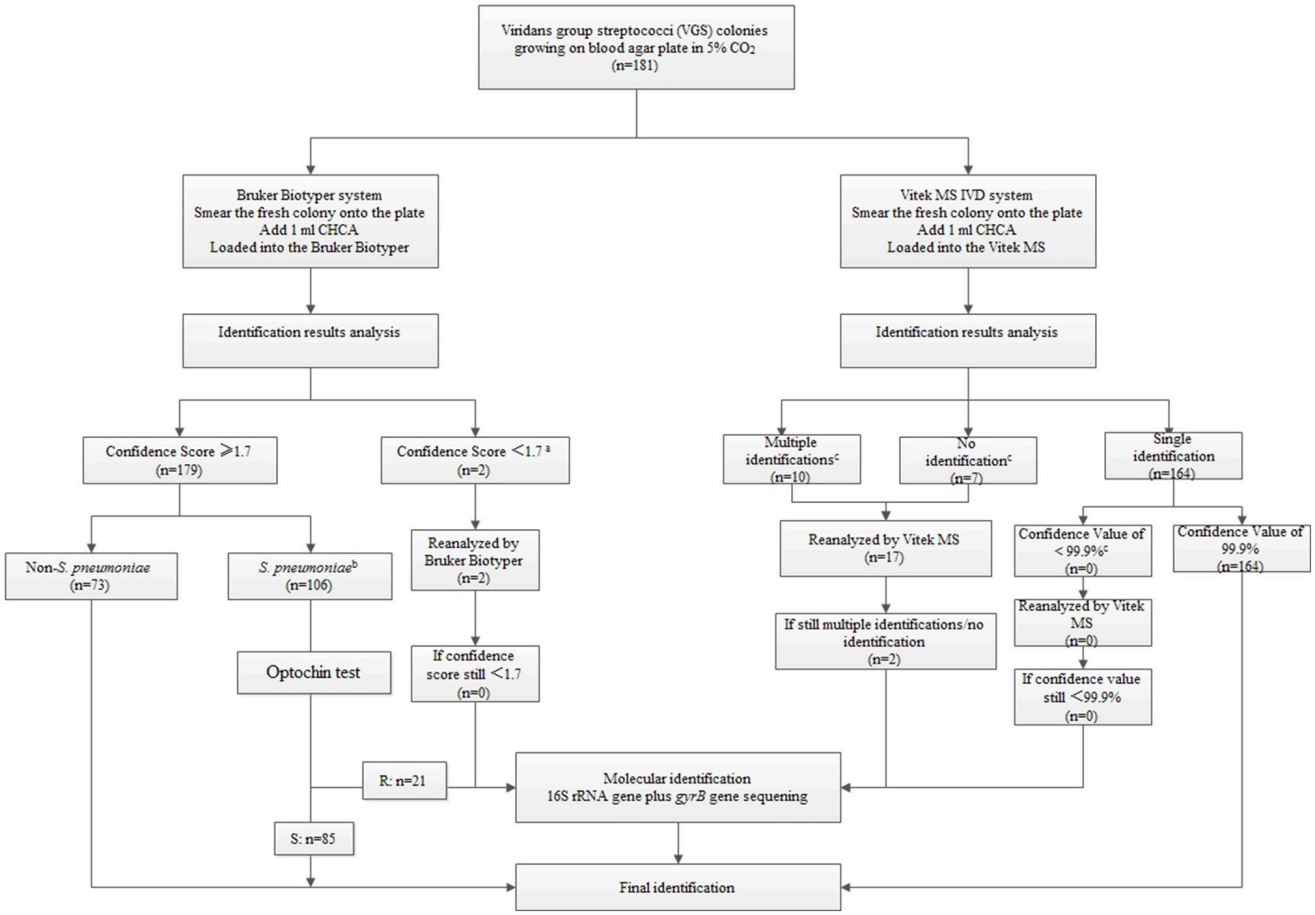
**An identification testing algorithm for viridans group streptococci (VGS) based on the Bruker Biotyper MS system/Vitek MS system and selective molecular identification (see Supplementary Tables S3 and S4)**. ^*a*^For Bruker Biotyper MS system, strains with identification scores <1.7 must be re-tested. ^*b*^Any identification of *S. pneumoniae* even with a score of >2.0, should be preliminatory and then do further sequencing for the high incorrect identification rate. Gene-based analysis confirmed that 85 out of the 106 isolates were *S. pneumoniae* while the rest are non-*S. pneumoniae* (Supplementary Table S3). ^*c*^For Vitek MS, initial identification results with a confidence value <99.9% for a single isolate or with multiple/no identification results, must be repeated (Supplementary Table S4).

Based on our findings, we designed an identification algorithm for the best way to identify species within the VGS group (Figure 2). Laboratories with either of the instruments could refer to this algorithm easily. For Bruker Biotyper MS system, strains with identification scores <1.7 must be re-tested since VGS are very homologous species. Any identification of *S. pneumoniae* even with a score of >2.0, should be discounted, optochin test and gene-based analysis need to be performed for confirmation. For Vitek MS, initial identification results with a confidence value <99.9% for a single result or with multiple/no identification results, must be repeated. If the repeat analysis fails to provide a high confidence value, these isolates should be identified by molecular methods.

Study limitations include possible selection bias as isolates were from a single center, imbalance in group/species distribution of isolates, with some groups and species poorly represented, e.g., the bovis and salivarius groups. And finally, for uniformity, protein extraction step was not performed for all assays.

## Summary

This study shows that both the Bruker Biotyper and the Vitek MS IVD systems can provide a good alternative to phenotypic methods for VGS identification. However, further improvements in the data bases are needed to increase the identification accuracy. In the mean-time, gene-based sequencing remains the best way to correctly identify VGS species. The proposed integrated algorithm is a practical approach in VGS identification at this stage.

## Author contributions

MZ, QY, and YX conceived and designed the experiments, performed the experiments, analyzed the data, and wrote the paper. TK and FK revised the paper critically for important intellectual content. LZ, MX, and YZ read and approved the final version of the manuscript.

## Funding

This work was supported by Research Special Fund for Public Welfare Industry of Health (Grant no. 201402001). The funders had no role in study design, data collection and analysis, decision to publish, or preparation of the manuscript.

### Conflict of interest statement

The authors declare that the research was conducted in the absence of any commercial or financial relationships that could be construed as a potential conflict of interest.

## Abbreviations

MALDI-TOF: matrix-assisted laser desorption ionization-time of flight
VGS: viridans group streptococci
MLSA: multilocus sequence analysis
PUMCH: Peking Union Medical College Hospital
IVD: *In Vitro* Diagnosis
RUO: Research Use Only
CI: confidence interval
PPV: positive predictive value
NPV: negative predictive value

## References

[B1] Angeletti S. Dicuonzo G. Avola A. Crea F. Dedej E. Vailati F.. (2015). Viridans Group Streptococci clinical isolates: MALDI-TOF mass spectrometry versus gene sequence-based identification. PLoS ONE 10:e0120502. 10.1371/journal.pone.0120502 25781023 PMC4362942

[B2] Arbique J. C. Poyart C. Trieu-Cuot P. Quesne G. Carvalho Mda G. Steigerwalt A. G.. (2004). Accuracy of phenotypic and genotypic testing for identification of *Streptococcus pneumoniae* and description of *Streptococcus pseudopneumoniae* sp. nov. J. Clin. Microbiol. 42, 4686–4696. 10.1128/JCM.42.10.4686-4696.2004 15472328 PMC522306

[B3] Bishop C. J. Aanensen D. M. Jordan G. E. Kilian M. Hanage W. P. Spratt B. G. (2009). Assigning strains to bacterial species via the internet. BMC Biol. 7:3. 10.1186/1741-7007-7-3 19171050 PMC2636762

[B4] Bizzini A. Durussel C. Bille J. Greub G. Prod'hom G. (2010). Performance of matrix-assisted laser desorption ionization-time of flight mass spectrometry for identification of bacterial strains routinely isolated in a clinical microbiology laboratory. J. Clin. Microbiol. 48, 1549–1554. 10.1128/JCM.01794-09 20220166 PMC2863943

[B5] Davies A. P. Reid M. Hadfield S. J. Johnston S. Mikhail J. Harris L. G.. (2012). Identification of clinical isolates of alpha-hemolytic streptococci by 16S rRNA gene sequencing, matrix-assisted laser desorption ionization-time of flight mass spectrometry using MALDI Biotyper, and conventional phenotypic methods: a comparison. J. Clin. Microbiol. 50, 4087–4090. 10.1128/JCM.02387-12 22993176 PMC3502998

[B6] Doern C. D. Burnham C. A. (2010). It's not easy being green: the viridans group streptococci, with a focus on pediatric clinical manifestations. J. Clin. Microbiol. 48, 3829–3835. 10.1128/JCM.01563-10 20810781 PMC3020876

[B7] Dubois D. Segonds C. Prere M. F. Marty N. Oswald E. (2013). Identification of clinical *Streptococcus pneumoniae* isolates among other alpha and nonhemolytic streptococci by use of the Vitek MS matrix-assisted laser desorption ionization-time of flight mass spectrometry system. J. Clin. Microbiol. 51, 1861–1867. 10.1128/JCM.03069-12 23576536 PMC3716079

[B8] Facklam R. (2002). What happened to the streptococci: overview of taxonomic and nomenclature changes. Clin. Microbiol. Rev. 15, 613–630. 10.1128/CMR.15.4.613-630.2002 12364372 PMC126867

[B9] Galloway-Peña J. Sahasrabhojane P. Tarrand J. Han X. Y. Shelburne S. A. (2014). GyrB polymorphisms accurately assign invasive viridans group streptococcal species. J. Clin. Microbiol. 52, 2905–2912. 10.1128/JCM.01068-14 24899021 PMC4136163

[B10] Haanperä M. Jalava J. Huovinen P. Meurman O. Rantakokko-Jalava K. (2007). Identification of alpha-hemolytic streptococci by pyrosequencing the 16S rRNA gene and by use of VITEK 2. J. Clin. Microbiol. 45, 762–770. 10.1128/JCM.01342-06. 17215341 PMC1829103

[B11] Ikryannikova L. N. Filimonova A. V. Malakhova M. V. Savinova T. Filimonova O. Ilina E. N.. (2013). Discrimination between *Streptococcus pneumoniae* and *Streptococcus mitis* based on sorting of their MALDI mass spectra. Clin. Microbiol. Infect. 19, 1066–1071. 10.1111/1469-0691.12113 23331578

[B12] Ikryannikova L. N. Lapin K. N. Malakhova M. V. Filimonova A. V. Ilina E. N. Dubovickaya V. A.. (2011). Misidentification of alpha-hemolytic streptococci by routine tests in clinical practice. Infect. Genet. Evol. 11, 1709–1715. 10.1016/j.meegid.2011.07.010 21798371

[B13] Ip M. Chi F. Chau S. S. Hui M. Tang J. Chan P. K. (2006). Use of the housekeeping genes, gdh (zwf) and gki, in multilocus sequence typing to differentiate *Streptococcus pneumoniae* from *Streptococcus mitis* and *Streptococcus oralis*. Diagn. Microbiol. Infect. Dis. 56, 321–324. 10.1016/j.diagmicrobio.2006.04.013 16765553

[B14] Isaksson J. Rasmussen M. Nilson B. Stadler L. S. Kurland S. Olaison L.. (2015). Comparison of species identification of endocarditis associated viridans streptococci using rnpB genotyping and 2 MALDI-TOF systems. Diagn. Microbiol. Infect. Dis. 81, 240–245. 10.1016/j.diagmicrobio.2014.12.007 25616316

[B15] Kärpänoja P. Harju I. Rantakokko-Jalava K. Haanperä M. Sarkkinen H. (2014). Evaluation of two matrix-assisted laser desorption ionization-time of flight mass spectrometry systems for identification of viridans group streptococci. Eur. J. Clin. Microbiol. Infect. Dis. 33, 779–788. 10.1007/s10096-013-2012-8 24202732

[B16] Kawamura Y. Hou X. G. Sultana F. Miura H. Ezaki T. (1995). Determination of 16S rRNA sequences of *Streptococcus mitis* and *Streptococcus gordonii* and phylogenetic relationships among members of the genus Streptococcus. Int. J. Syst. Bacteriol. 45, 406–408. 10.1099/00207713-45-2-406 7537076

[B17] Konishi I. Hoshino T. Kondo Y. Saito K. Nishiguchi M. Sato K.. (2009). Phylogenetic analyses and detection of viridans streptococci based on sequences and denaturing gradient gel electrophoresis of the rod shape-determining protein gene. J. Oral. Microbiol. 1. 10.3402/jom.v1i0.2015 21523207 PMC3077002

[B18] López Roa P. Sanchez Carrillo C. Marín M. Romero F. Cercenado E. Bouza E. (2013). Value of matrix-assisted laser desorption ionization-time of flight for routine identification of viridans group streptococci causing bloodstream infections. Clin. Microbiol. Infect. 19, 438–444. 10.1111/j.1469-0691.2012.03837.x 22510157

[B19] Maeda Y. Murayama M. Goldsmith C. E. Coulter W. A. Mason C. Millar B. C.. (2011). Molecular characterization and phylogenetic analysis of quinolone resistance-determining regions (QRDRs) of gyrA, gyrB, parC and parE gene loci in viridans group streptococci isolated from adult patients with cystic fibrosis. J. Antimicrob. Chemother. 66, 476–486. 10.1093/jac/dkq485 21193474

[B20] Neville S. A. Lecordier A. Ziochos H. Chater M. J. Gosbell I. B. Maley M. W.. (2011). Utility of matrix-assisted laser desorption ionization-time of flight mass spectrometry following introduction for routine laboratory bacterial identification. J. Clin. Microbiol. 49, 2980–2984. 10.1128/JCM.00431-11 21632894 PMC3147730

[B21] Nielsen X. C. Justesen U. S. Dargis R. Kemp M. Christensen J. J. (2009). Identification of clinically relevant nonhemolytic Streptococci on the basis of sequence analysis of 16S-23S intergenic spacer region and partial gdh gene. J. Clin. Microbiol. 47, 932–939. 10.1128/JCM.01449-08 19193846 PMC2668326

[B22] Park H. K. Yoon J. W. Shin J. W. Kim J. Y. Kim W. (2010). rpoA is a useful gene for identification and classification of *Streptococcus pneumoniae* from the closely related viridans group streptococci. FEMS Microbiol. Lett. 305, 58–64. 10.1111/j.1574-6968.2010.01913.x 20158524

[B23] Poyart C. Quesne G. Coulon S. Berche P. Trieu-Cuot P. (1998). Identification of streptococci to species level by sequencing the gene encoding the manganese-dependent superoxide dismutase. J. Clin. Microbiol. 36, 41–47. 9431917 10.1128/jcm.36.1.41-47.1998 PMC124804

[B24] Rychert J. Burnham C. A. Bythrow M. Garner O. B. Ginocchio C. C. Jennemann R.. (2013). Multicenter evaluation of the Vitek MS matrix-assisted laser desorption ionization-time of flight mass spectrometry system for identification of Gram-positive aerobic bacteria. J. Clin. Microbiol. 51, 2225–2231. 10.1128/JCM.00682-13 23658261 PMC3697712

[B25] Scholz C. F. Poulsen K. Kilian M. (2012). Novel molecular method for identification of *Streptococcus pneumoniae* applicable to clinical microbiology and 16S rRNA sequence-based microbiome studies. J. Clin. Microbiol. 50, 1968–1973. 10.1128/JCM.00365-12 22442329 PMC3372098

[B26] Seng P. Drancourt M. Gouriet F. La Scola B. Fournier P. E. Rolain J. M.. (2009). Ongoing revolution in bacteriology: routine identification of bacteria by matrix-assisted laser desorption ionization time-of-flight mass spectrometry. Clin. Infect. Dis. 49, 543–551. 10.1086/600885 19583519

[B27] Sinner S. Tunkel A. (2009). Viridans streptococci, groups C and Gstreptococci, and Gemella species, in Mandell, Douglas, and Bennett's Principles and Practice of Infectious Diseases, 7th Edn., eds Mandell G. L. Bennett J. E. Dolin R. (Philadelphia, PA: Churchill Livingstone Elsevier), 2667–2680.

[B28] Spellberg B. Brandt C. (2011). Streptococcus, in Manual of Clinical Microbiology, 10th Edn., eds Versalovic J. Carroll K. C. Funke G. Jorgensen J. H. Landry M. L. Warnock D. W. (Washington, DC: ASM Press), 331–349.

[B29] Suzuki N. Seki M. Nakano Y. Kiyoura Y. Maeno M. Yamashita Y. (2005). Discrimination of *Streptococcus pneumoniae* from viridans group streptococci by genomic subtractive hybridization. J. Clin. Microbiol. 43, 4528–4534. 10.1128/JCM.43.9.4528-4534.2005 16145102 PMC1234109

[B30] Teles C. Smith A. Ramage G. Lang S. (2011). Identification of clinically relevant viridans group streptococci by phenotypic and genotypic analysis. Eur. J. Clin. Microbiol. Infect. Dis. 30, 243–250. 10.1007/s10096-010-1076-y 20981464

[B31] Welker M. (2011). Proteomics for routine identification of microorganisms. Proteomics 11, 3143–3153. 10.1002/pmic.201100049 21726051

[B32] Wessels E. Schelfaut J. J. Bernards A. T. Claas E. C. (2012). Evaluation of several biochemical and molecular techniques for identification of *Streptococcus pneumoniae* and *Streptococcus pseudopneumoniae* and their detection in respiratory samples. J. Clin. Microbiol. 50, 1171–1177. 10.1128/JCM.06609-11 22278834 PMC3318541

[B33] Westling K. Julander I. Ljungman P. Vondracek M. Wretlind B. Jalal S. (2008). Identification of species of viridans group streptococci in clinical blood culture isolates by sequence analysis of the RNase P RNA gene, rnpB. J. Infect. 56, 204–210. 10.1016/j.jinf.2007.12.006 18255158

