# Supplementary material for: Using Matrix-Assisted Laser Desorption Ionization-Time of Flight (MALDI-TOF) Complemented with Selected 16S rRNA and gyrB Genes Sequencing to Practically Identify Clinical Important Viridans Group Streptococci (VGS)
Source: Front Microbiol. 2016 Aug 26;7:1328. doi: 10.3389/fmicb.2016.01328 (PMC5000867; doi:10.3389/fmicb.2016.01328)
Supplement: Supplementary file 1 [file Table1.DOCX]

**Using matrix-assisted laser desorption ionization-time of flight (MALDI-TOF) complemented with selected 16S rRNA and *gyrB* genes sequencing to practically identify clinical important viridans group streptococci (VGS)**

Menglan Zhou, Qiwen Yang^*^, Timothy Kudinha, Li Zhang, Meng Xiao, Fanrong Kong, Yupei Zhao, Ying-Chun Xu^*^

^*^**Correspondence:** Qiwen Yang: yangqiwen81@163.com**,** YingChun Xu: xycpumch@139.com

**Supplementary Table S1. Sample sources of 181 viridans group streptococci (VGS) isolates used in this study.**

| **Reference Identification** | **No.**  **of isolates** | **No. of isolates from different sample sources** | | | | | | | | | | | | | | | | | | | | |
| --- | --- | --- | --- | --- | --- | --- | --- | --- | --- | --- | --- | --- | --- | --- | --- | --- | --- | --- | --- | --- | --- | --- |
|  |  | **A** | **B** | **BLF** | **CSS** | **CS** | **CSF** | **DF** | **IS** | **LNB** | **MU** | **OBF** | **OS** | **PE** | **PDF** | **PS** | **PF** | **Se** | **Sp** | **T** | **TA** | **VS** |
| **Mitis group** | **107** | **3** | **8** | **2** | **1** | **1** | **4** | **1** | **0** | **0** | **0** | **0** | **1** | **0** | **0** | **1** | **3** | **1** | **71** | **1** | **9** | **0** |
| *S.mitis* | 11 | 1 | 3 | 0 | 0 | 0 | 0 | 0 | 0 | 0 | 0 | 0 | 1 | 0 | 0 | 0 | 3 | 1 | 0 | 1 | 1 | 0 |
| *S.oralis* | 2 | 0 | 0 | 0 | 1 | 0 | 0 | 1 | 0 | 0 | 0 | 0 | 0 | 0 | 0 | 0 | 0 | 0 | 0 | 0 | 0 | 0 |
| *S.pseudopneumoniae* | 9 | 0 | 0 | 0 | 0 | 0 | 0 | 0 | 0 | 0 | 0 | 0 | 0 | 0 | 0 | 0 | 0 | 0 | 8 | 0 | 1 | 0 |
| *S.pneumoniae* | 85 | 2 | 5 | 2 | 0 | 1 | 4 | 0 | 0 | 0 | 0 | 0 | 0 | 0 | 0 | 1 | 0 | 0 | 63 | 0 | 7 | 0 |
| **Anginosus group** | **52** | **5** | **7** | **4** | **0** | **0** | **0** | **4** | **2** | **1** | **9** | **1** | **2** | **2** | **0** | **1** | **0** | **0** | **10** | **0** | **2** | **2** |
| *S.anginosus* | 29 | 4 | 5 | 1 | 0 | 0 | 0 | 1 | 1 | 0 | 9 | 0 | 2 | 1 | 0 | 0 | 0 | 0 | 4 | 0 | 0 | 1 |
| *S.constellatus* | 19 | 1 | 2 | 3 | 0 | 0 | 0 | 2 | 0 | 1 | 0 | 1 | 0 | 0 | 0 | 0 | 0 | 0 | 6 | 0 | 2 | 1 |
| *S.intermedius* | 4 | 0 | 0 | 0 | 0 | 0 | 0 | 1 | 1 | 0 | 0 | 0 | 0 | 1 | 0 | 1 | 0 | 0 | 0 | 0 | 0 | 0 |
| **Sanguinis group** | **12** | **0** | **11** | **0** | **0** | **0** | **0** | **0** | **0** | **0** | **0** | **0** | **0** | **0** | **0** | **0** | **0** | **0** | **0** | **1** | **0** | **0** |
| *S.sanguinis* | 8 | 0 | 7 | 0 | 0 | 0 | 0 | 0 | 0 | 0 | 0 | 0 | 0 | 0 | 0 | 0 | 0 | 0 | 0 | 1 | 0 | 0 |
| *S.gordonii* | 4 | 0 | 4 | 0 | 0 | 0 | 0 | 0 | 0 | 0 | 0 | 0 | 0 | 0 | 0 | 0 | 0 | 0 | 0 |  | 0 | 0 |
| **Salivarius group** | **2** | **0** | **0** | **0** | **0** | **0** | **0** | **0** | **0** | **0** | **0** | **0** | **0** | **0** | **1** | **0** | **0** | **0** | **0** | **1** | **0** | **0** |
| *S.salivarius* | 2 | 0 | 0 | 0 | 0 | 0 | 0 | 0 | 0 | 0 | 0 | 0 | 0 | 0 | 1 | 0 | 0 | 0 | 0 | 1 | 0 | 0 |
| **Bovis group** | **8** | **0** | **3** | **0** | **0** | **0** | **0** | **1** | **0** | **0** | **0** | **0** | **0** | **0** | **3** | **0** | **0** | **0** | **0** | **0** | **0** | **1** |
| *S.lutetiensis* | 2 | 0 | 0 | 0 | 0 | 0 | 0 | 0 | 0 | 0 | 0 | 0 | 0 | 0 | 2 | 0 | 0 | 0 | 0 | 0 | 0 | 0 |
| *S.gallolyticus* | 6 | 0 | 3 | 0 | 0 | 0 | 0 | 1 | 0 | 0 | 0 | 0 | 0 | 0 | 1 | 0 | 0 | 0 | 0 | 0 | 0 | 1 |
| **Overall** | **181** | **8** | **29** | **6** | **1** | **1** | **4** | **6** | **2** | **1** | **9** | **1** | **3** | **2** | **4** | **2** | **3** | **1** | **81** | **3** | **11** | **3** |

Notes and abbreviations:

A: abscess; B: blood; BLF: bronchoalveolar lavage fluid; CSS: conjunctival sac secretions; CS: corneal secretions; CSF: cerebrospinal Fluid; DF: drainage fluid; IS: incision secretions; LNB: lymph node biopsies; MU: midstream urine; OBF: other body fluids; OS: other swabs; PE: pelvic effusion; PDF: peritoneal dialysis fluid; PS: pharyngeal swabs; PF: pleural fluids; Se: semen; Sp: sputum; T: tissue; TA: tracheal aspirates; VS: vaginal swabs
