# Supplementary material for: Using Matrix-Assisted Laser Desorption Ionization-Time of Flight (MALDI-TOF) Complemented with Selected 16S rRNA and gyrB Genes Sequencing to Practically Identify Clinical Important Viridans Group Streptococci (VGS)
Source: Front Microbiol. 2016 Aug 26;7:1328. doi: 10.3389/fmicb.2016.01328 (PMC5000867; doi:10.3389/fmicb.2016.01328)
Supplement: Supplementary file 2 [file Table2.DOCX]

**Using matrix-assisted laser desorption ionization-time of flight (MALDI-TOF) complemented with selected 16S rRNA and *gyrB* genes sequencing to practically identify clinical important viridans group streptococci (VGS)**

Menglan Zhou, Qiwen Yang^*^, Timothy Kudinha, Li Zhang, Meng Xiao, Fanrong Kong, Yupei Zhao, Ying-Chun Xu^*^

^*^**Correspondence:** Qiwen Yang: yangqiwen81@163.com**,** YingChun Xu: xycpumch@139.com

**Supplementary Table S2. Species and GenBank accession numbers of 16S rRNA gene and *gyrB* gene sequences for 181 viridans group streptococci (VGS) studied.**

| **Strain** | **Species** | **GenBank Accession Numbers** | |
| --- | --- | --- | --- |
|  |  | **16S rRNA (1251 bp)** | ***gyrB* (430 bp)** |
| PUA001 | *Streptococcus anginosus* | KX661043 | KX661224 |
| PUA002 | *Streptococcus anginosus* | KX661044 | KX661225 |
| PUA003 | *Streptococcus sanguinis* | KX661045 | KX661226 |
| PUA004 | *Streptococcus anginosus* | KX661046 | KX661227 |
| PUA005 | *Streptococcus anginosus* | KX661047 | KX661228 |
| PUA006 | *Streptococcus anginosus* | KX661048 | KX661229 |
| PUA007 | *Streptococcus anginosus* | KX661049 | KX661230 |
| PUA008 | *Streptococcus anginosus* | KX661050 | KX661231 |
| PUA009 | *Streptococcus anginosus* | KX661051 | KX661232 |
| PUA010 | *Streptococcus anginosus* | KX661052 | KX661233 |
| PUA011 | *Streptococcus mitis* | KX661053 | KX661234 |
| PUA012 | *Streptococcus anginosus* | KX661054 | KX661235 |
| PUA013 | *Streptococcus anginosus* | KX661055 | KX661236 |
| PUA014 | *Streptococcus anginosus* | KX661056 | KX661237 |
| PUA015 | *Streptococcus anginosus* | KX661057 | KX661238 |
| PUA016 | *Streptococcus anginosus* | KX661058 | KX661239 |
| PUA017 | *Streptococcus anginosus* | KX661059 | KX661240 |
| PUA018 | *Streptococcus mitis* | KX661060 | KX661241 |
| PUA019 | *Streptococcus anginosus* | KX661061 | KX661242 |
| PUA020 | *Streptococcus anginosus* | KX661062 | KX661243 |
| PUA021 | *Streptococcus anginosus* | KX661063 | KX661244 |
| PUA022 | *Streptococcus constellatus* | KX661064 | KX661245 |
| PUA023 | *Streptococcus anginosus* | KX661065 | KX661246 |
| PUA024 | *Streptococcus salivarius* | KX661066 | KX661247 |
| PUA025 | *Streptococcus anginosus* | KX661067 | KX661248 |
| PUA026 | *Streptococcus anginosus* | KX661068 | KX661249 |
| PUA027 | *Streptococcus constellatus* | KX661069 | KX661250 |
| PUA028 | *Streptococcus anginosus* | KX661070 | KX661251 |
| PUA029 | *Streptococcus anginosus* | KX661071 | KX661252 |
| PUA030 | *Streptococcus anginosus* | KX661072 | KX661253 |
| PUA031 | *Streptococcus anginosus* | KX661073 | KX661254 |
| PUA032 | *Streptococcus anginosus* | KX661074 | KX661255 |
| PUA033 | *Streptococcus anginosus* | KX661075 | KX661256 |
| PUA034 | *Streptococcus constellatus* | KX661076 | KX661257 |
| PUA035 | *Streptococcus constellatus* | KX661077 | KX661258 |
| PUA036 | *Streptococcus constellatus* | KX661078 | KX661259 |
| PUA037 | *Streptococcus constellatus* | KX661079 | KX661260 |
| PUA038 | *Streptococcus lutetiensis* | KX661080 | KX661261 |
| PUA039 | *Streptococcus constellatus* | KX661081 | KX661262 |
| PUA040 | *Streptococcus constellatus* | KX661082 | KX661263 |
| PUA041 | *Streptococcus constellatus* | KX661083 | KX661264 |
| PUA042 | *Streptococcus constellatus* | KX661084 | KX661265 |
| PUA043 | *Streptococcus constellatus* | KX661085 | KX661266 |
| PUA044 | *Streptococcus sanguinis* | KX661086 | KX661267 |
| PUA045 | *Streptococcus constellatus* | KX661087 | KX661268 |
| PUA046 | *Streptococcus constellatus* | KX661088 | KX661269 |
| PUA047 | *Streptococcus anginosus* | KX661089 | KX661270 |
| PUA048 | *Streptococcus gordonii* | KX661090 | KX661271 |
| PUA049 | *Streptococcus constellatus* | KX661091 | KX661272 |
| PUA050 | *Streptococcus constellatus* | KX661092 | KX661273 |
| PUA051 | *Streptococcus constellatus* | KX661093 | KX661274 |
| PUA052 | *Streptococcus constellatus* | KX661094 | KX661275 |
| PUA053 | *Streptococcus constellatus* | KX661095 | KX661276 |
| PUA054 | *Streptococcus constellatus* | KX661096 | KX661277 |
| PUA055 | *Streptococcus mitis* | KX661097 | KX661278 |
| PUA056 | *Streptococcus mitis* | KX661098 | KX661279 |
| PUA057 | *Streptococcus mitis* | KX661099 | KX661280 |
| PUA058 | *Streptococcus mitis* | KX661100 | KX661281 |
| PUA059 | *Streptococcus mitis* | KX661101 | KX661282 |
| PUA060 | *Streptococcus gordonii* | KX661102 | KX661283 |
| PUA061 | *Streptococcus mitis* | KX661103 | KX661284 |
| PUA062 | *Streptococcus lutetiensis* | KX661104 | KX661285 |
| PUA063 | *Streptococcus oralis* | KX661105 | KX661286 |
| PUA064 | *Streptococcus mitis* | KX661106 | KX661287 |
| PUA065 | *Streptococcus gallolyticus* | KX661107 | KX661288 |
| PUA066 | *Streptococcus gallolyticus* | KX661108 | KX661289 |
| PUA067 | *Streptococcus gallolyticus* | KX661109 | KX661290 |
| PUA068 | *Streptococcus gallolyticus* | KX661110 | KX661291 |
| PUA069 | *Streptococcus gallolyticus* | KX661111 | KX661292 |
| PUA070 | *Streptococcus gallolyticus* | KX661112 | KX661293 |
| PUA071 | *Streptococcus anginosus* | KX661113 | KX661294 |
| PUA072 | *Streptococcus sanguinis* | KX661114 | KX661295 |
| PUA073 | *Streptococcus sanguinis* | KX661115 | KX661296 |
| PUA074 | *Streptococcus sanguinis* | KX661116 | KX661297 |
| PUA075 | *Streptococcus sanguinis* | KX661117 | KX661298 |
| PUA076 | *Streptococcus sanguinis* | KX661118 | KX661299 |
| PUA077 | *Streptococcus oralis* | KX661119 | KX661300 |
| PUA078 | *Streptococcus intermedius* | KX661120 | KX661301 |
| PUA079 | *Streptococcus intermedius* | KX661121 | KX661302 |
| PUA080 | *Streptococcus intermedius* | KX661122 | KX661303 |
| PUA081 | *Streptococcus intermedius* | KX661123 | KX661304 |
| PUA082 | *Streptococcus salivarius* | KX661124 | KX661305 |
| PUA083 | *Streptococcus sanguinis* | KX661125 | KX661306 |
| PUA084 | *Streptococcus gordonii* | KX661126 | KX661307 |
| PUA085 | *Streptococcus gordonii* | KX661127 | KX661308 |
| PUA086 | *Streptococcus mitis* | KX661128 | KX661309 |
| PUA087 | *Streptococcus mitis* | KX661129 | KX661310 |
| PUA088 | *Streptococcus pseudopneumoniae* | KX661130 | KX661311 |
| PUA089 | *Streptococcus pseudopneumoniae* | KX661131 | KX661312 |
| PUA090 | *Streptococcus pseudopneumoniae* | KX661132 | KX661313 |
| PUA091 | *Streptococcus pseudopneumoniae* | KX661133 | KX661314 |
| PUA092 | *Streptococcus pseudopneumoniae* | KX661134 | KX661315 |
| PUA093 | *Streptococcus pseudopneumoniae* | KX661135 | KX661316 |
| PUA094 | *Streptococcus pseudopneumoniae* | KX661136 | KX661317 |
| PUA095 | *Streptococcus pseudopneumoniae* | KX661137 | KX661318 |
| PUA096 | *Streptococcus pseudopneumoniae* | KX661138 | KX661319 |
| PUS001 | *Streptococcus pneumoniae* | KX661139 |  |
| PUS002 | *Streptococcus pneumoniae* | KX661140 |  |
| PUS003 | *Streptococcus pneumoniae* | KX661141 |  |
| PUS004 | *Streptococcus pneumoniae* | KX661142 |  |
| PUS005 | *Streptococcus pneumoniae* | KX661143 |  |
| PUS006 | *Streptococcus pneumoniae* | KX661144 |  |
| PUS007 | *Streptococcus pneumoniae* | KX661145 |  |
| PUS008 | *Streptococcus pneumoniae* | KX661146 |  |
| PUS009 | *Streptococcus pneumoniae* | KX661147 |  |
| PUS010 | *Streptococcus pneumoniae* | KX661148 |  |
| PUS011 | *Streptococcus pneumoniae* | KX661149 |  |
| PUS012 | *Streptococcus pneumoniae* | KX661150 |  |
| PUS013 | *Streptococcus pneumoniae* | KX661151 |  |
| PUS014 | *Streptococcus pneumoniae* | KX661152 |  |
| PUS015 | *Streptococcus pneumoniae* | KX661153 |  |
| PUS016 | *Streptococcus pneumoniae* | KX661154 |  |
| PUS017 | *Streptococcus pneumoniae* | KX661155 |  |
| PUS018 | *Streptococcus pneumoniae* | KX661156 |  |
| PUS019 | *Streptococcus pneumoniae* | KX661157 |  |
| PUS020 | *Streptococcus pneumoniae* | KX661158 |  |
| PUS021 | *Streptococcus pneumoniae* | KX661159 |  |
| PUS022 | *Streptococcus pneumoniae* | KX661160 |  |
| PUS023 | *Streptococcus pneumoniae* | KX661161 |  |
| PUS024 | *Streptococcus pneumoniae* | KX661162 |  |
| PUS025 | *Streptococcus pneumoniae* | KX661163 |  |
| PUS026 | *Streptococcus pneumoniae* | KX661164 |  |
| PUS027 | *Streptococcus pneumoniae* | KX661165 |  |
| PUS028 | *Streptococcus pneumoniae* | KX661166 |  |
| PUS029 | *Streptococcus pneumoniae* | KX661167 |  |
| PUS030 | *Streptococcus pneumoniae* | KX661168 |  |
| PUS031 | *Streptococcus pneumoniae* | KX661169 |  |
| PUS032 | *Streptococcus pneumoniae* | KX661170 |  |
| PUS033 | *Streptococcus pneumoniae* | KX661171 |  |
| PUS034 | *Streptococcus pneumoniae* | KX661172 |  |
| PUS035 | *Streptococcus pneumoniae* | KX661173 |  |
| PUS036 | *Streptococcus pneumoniae* | KX661174 |  |
| PUS037 | *Streptococcus pneumoniae* | KX661175 |  |
| PUS038 | *Streptococcus pneumoniae* | KX661176 |  |
| PUS039 | *Streptococcus pneumoniae* | KX661177 |  |
| PUS040 | *Streptococcus pneumoniae* | KX661178 |  |
| PUS041 | *Streptococcus pneumoniae* | KX661179 |  |
| PUS042 | *Streptococcus pneumoniae* | KX661180 |  |
| PUS043 | *Streptococcus pneumoniae* | KX661181 |  |
| PUS044 | *Streptococcus pneumoniae* | KX661182 |  |
| PUS045 | *Streptococcus pneumoniae* | KX661183 |  |
| PUS046 | *Streptococcus pneumoniae* | KX661184 |  |
| PUS047 | *Streptococcus pneumoniae* | KX661185 |  |
| PUS048 | *Streptococcus pneumoniae* | KX661186 |  |
| PUS049 | *Streptococcus pneumoniae* | KX661187 |  |
| PUS050 | *Streptococcus pneumoniae* | KX661188 |  |
| PUS051 | *Streptococcus pneumoniae* | KX661189 |  |
| PUS052 | *Streptococcus pneumoniae* | KX661190 |  |
| PUS053 | *Streptococcus pneumoniae* | KX661191 |  |
| PUS054 | *Streptococcus pneumoniae* | KX661192 |  |
| PUS055 | *Streptococcus pneumoniae* | KX661193 |  |
| PUS056 | *Streptococcus pneumoniae* | KX661194 |  |
| PUS057 | *Streptococcus pneumoniae* | KX661195 |  |
| PUS058 | *Streptococcus pneumoniae* | KX661196 |  |
| PUS059 | *Streptococcus pneumoniae* | KX661197 |  |
| PUS060 | *Streptococcus pneumoniae* | KX661198 |  |
| PUS061 | *Streptococcus pneumoniae* | KX661199 |  |
| PUS062 | *Streptococcus pneumoniae* | KX661200 |  |
| PUS063 | *Streptococcus pneumoniae* | KX661201 |  |
| PUS064 | *Streptococcus pneumoniae* | KX661202 |  |
| PUS065 | *Streptococcus pneumoniae* | KX661203 |  |
| PUS066 | *Streptococcus pneumoniae* | KX661204 |  |
| PUS067 | *Streptococcus pneumoniae* | KX661205 |  |
| PUS068 | *Streptococcus pneumoniae* | KX661206 |  |
| PUS069 | *Streptococcus pneumoniae* | KX661207 |  |
| PUS070 | *Streptococcus pneumoniae* | KX661208 |  |
| PUS071 | *Streptococcus pneumoniae* | KX661209 |  |
| PUS072 | *Streptococcus pneumoniae* | KX661210 |  |
| PUS073 | *Streptococcus pneumoniae* | KX661211 |  |
| PUS074 | *Streptococcus pneumoniae* | KX661212 |  |
| PUS075 | *Streptococcus pneumoniae* | KX661213 |  |
| PUS076 | *Streptococcus pneumoniae* | KX661214 |  |
| PUS077 | *Streptococcus pneumoniae* | KX661215 |  |
| PUS078 | *Streptococcus pneumoniae* | KX661216 |  |
| PUS079 | *Streptococcus pneumoniae* | KX661217 |  |
| PUS080 | *Streptococcus pneumoniae* | KX661218 |  |
| PUS081 | *Streptococcus pneumoniae* | KX661219 |  |
| PUS082 | *Streptococcus pneumoniae* | KX661220 |  |
| PUS083 | *Streptococcus pneumoniae* | KX661221 |  |
| PUS084 | *Streptococcus pneumoniae* | KX661222 |  |
| PUS085 | *Streptococcus pneumoniae* | KX661223 |  |
