# Supplementary material for: Using Matrix-Assisted Laser Desorption Ionization-Time of Flight (MALDI-TOF) Complemented with Selected 16S rRNA and gyrB Genes Sequencing to Practically Identify Clinical Important Viridans Group Streptococci (VGS)
Source: Front Microbiol. 2016 Aug 26;7:1328. doi: 10.3389/fmicb.2016.01328 (PMC5000867; doi:10.3389/fmicb.2016.01328)
Supplement: Supplementary file 3 [file Table3.DOCX]

**Using matrix-assisted laser desorption ionization-time of flight (MALDI-TOF) complemented with selected 16S rRNA and *gyrB* genes sequencing to practically identify clinical important viridans group streptococci (VGS)**

Menglan Zhou, Qiwen Yang^*^, Timothy Kudinha, Li Zhang, Meng Xiao, Fanrong Kong, Yupei Zhao, Ying-Chun Xu^*^

^*^**Correspondence:** Qiwen Yang: yangqiwen81@163.com**,** YingChun Xu: xycpumch@139.com

**Supplementary Table S3. Performance of the Bruker Biotyper system for the identification of 181 viridans group streptococci (VGS)**

**isolates comparing with reference 16S rRNA gene and *gyrB* genes sequencing identification assays.**

| **Reference Identification** | **No. (%) of isolates** | **No. (%) of isolates with Bruker Biotyper MS results of:** | | | |
| --- | --- | --- | --- | --- | --- |
|  |  | **Correct identification to species level  (single result)** | **Correct identification to groups level (multiple results)** | **Misidentification (single/multiple results)** | **No identification** |
| **Mitis group** | **107** | **86 (80.4%)** | **0 (0)** | **21 (19.6%)** | **0 (0)** |
| *S. mitis* | 11 | 0 (0) | 0 (0) | 11 (100%) | 0 (0) |
| *S. oralis* | 2 | 0 (0) | 0 (0) | 2 (100%) | 0 (0) |
| *S. pseudopneumoniae* | 9 | 1 (11.1%) | 0 (0) | 8 (88.9%) | 0 (0) |
| *S. pneumoniae* | 85 | 85 (100%) | 0 (0) | 0 (0) | 0 (0) |
| **Anginosus group** | **52** | **52 (100%)** | **0 (0)** | **0 (0)** | **0 (0)** |
| *S. anginosus* | 29 | 29 (100%) | 0 (0) | 0 (0) | 0 (0) |
| *S. constellatus* | 19 | 19 (100%) | 0 (0) | 0 (0) | 0 (0) |
| *S. intermedius* | 4 | 4 (100%) | 0 (0) | 0 (0) | 0 (0) |
| **Sanguinis group** | **12** | **12 (100%)** | **0 (0)** | **0 (0)** | **0 (0)** |
| *S. sanguinis* | 8 | 8 (100%) | 0 (0) | 0 (0) | 0 (0) |
| *S. gordonii* | 4 | 4 (100%) | 0 (0) | 0 (0) | 0 (0) |
| **Salivarius group** | **2** | **2 (100%)** | **0 (0)** | **0 (0)** | **0 (0)** |
| *S. salivarius* | 2 | 2 (100%) | 0 (0) | 0 (0) | 0 (0) |
| **Bovis group** | **8** | **8 (100%)** | **0 (0)** | **0 (0)** | **0 (0)** |
| *S. lutetiensis* | 2 | 2 (100%) | 0 (0) | 0 (0) | 0 (0) |
| *S. gallolyticus* | 6 | 6 (100%) | 0 (0) | 0 (0) | 0 (0) |
| **Overall** | **181** | **160 (88.4%)** | **0 (0))** | **21 (11.6%)** | **0 (0)** |
