# Supplementary material for: Using Matrix-Assisted Laser Desorption Ionization-Time of Flight (MALDI-TOF) Complemented with Selected 16S rRNA and gyrB Genes Sequencing to Practically Identify Clinical Important Viridans Group Streptococci (VGS)
Source: Front Microbiol. 2016 Aug 26;7:1328. doi: 10.3389/fmicb.2016.01328 (PMC5000867; doi:10.3389/fmicb.2016.01328)
Supplement: Supplementary file 5 [file Table5.DOCX]

**Using matrix-assisted laser desorption ionization-time of flight (MALDI-TOF) complemented with selected 16S rRNA and *gyrB* genes sequencing to practically identify clinical important viridans group streptococci (VGS)**

Menglan Zhou, Qiwen Yang^*^, Timothy Kudinha, Li Zhang, Meng Xiao, Fanrong Kong, Yupei Zhao, Ying-Chun Xu^*^

^*^**Correspondence:** Qiwen Yang: yangqiwen81@163.com**,** YingChun Xu: xycpumch@139.com

**Supplementary Table S5. Performance of the Vitek MS RUO system for the identification of 181 viridans group streptococci (VGS)**

**isolates comparing with reference 16S rRNA gene and *gyrB* genes sequencing identification assays.**

| **Reference Identification** | **No. (%) of isolates** | **No. (%) of isolates with Vitek MS RUO results of:** | | | | |
| --- | --- | --- | --- | --- | --- | --- |
|  |  | **Correct identification to species level  (single result)** | **Correct identification to group level  (multiple results)** | **Correct identification to genus level  (single/multiple results)** | **Misidentification  (single/multiple results)** | **No identification** |
| **Mitis group** | **107** | **17 (15.9%)** | **74 (69.2%)** | **9 (8.4%)** | **3 (2.8%)** | **4 (3.7%)** |
| *S. mitis* | 11 | 0 (0) | 8 (72.7%) | 1 (9.1%) | 2 (18.2%) | 0 (0) |
| *S. oralis* | 2 | 0 (0) | 1 (50%) | 0 (0) | 0 (0) | 1 (50%) |
| *S. pseudopneumoniae* | 9 | 0 (0) | 6 (66.7%) | 2 (22.2%) | 1 (11.1%) | 0 (0) |
| *S. pneumoniae* | 85 | 17 (20%) | 59 (69.4%) | 6 (7.1%) | 0 (0) | 3 (3.5%) |
| **Anginosus group** | **52** | **38 (73.1%)** | **0 (0)** | **0 (0)** | **13 (25%)** | **1 (1.9%)** |
| *S. anginosus* | 29 | 29 (100%) | 0 (0) | 0 (0) | 0 (0) | 0 (0) |
| *S. constellatus* | 19 | 5 (26.3%) | 0 (0) | 0 (0) | 13 (68.4%) | 1 (5.3%) |
| *S. intermedius* | 4 | 4 (100%) | 0 (0) | 0 (0) | 0 (0) | 0 (0) |
| **Sanguinis group** | **12** | **11 (91.7%)** | **0 (0)** | **0 (0)** | **0 (0)** | **1 (8.3%)** |
| *S. sanguinis* | 8 | 7 (87.5%) | 0 (0) | 0 (0) | 0 (0) | 1 (12.5%) |
| *S. gordonii* | 4 | 4 (100%) | 0 (0) | 0 (0) | 0 (0) | 0 (0) |
| **Salivarius group** | **2** | **2 (100%)** | **0 (0)** | **0 (0)** | **0 (0)** | **0 (0)** |
| *S. salivarius* | 2 | 2 (100%) | 0 (0) | 0 (0) | 0 (0) | 0 (0) |
| **Bovis group** | **8** | **2 (25%)** | **2 (25%)** | **4 (50%)** | **0 (0)** | **0 (0)** |
| *S. lutetiensis* | 2 | 0 (0) | 2 (100%) | 0 (0) | 0 (0) | 0 (0) |
| *S. gallolyticus* | 6 | 2 (33.3%) | 0 (0) | 4 (66.7%) | 0 (0) | 0 (0) |
| **Overall** | **181** | **70 (38.7%)** | **76 (42.0%)** | **13 (7.2%)** | **16 (8.8%)** | **6 (3.3%)** |
